# Progress of the Use of Chloroform

**Published:** 1848-04

**Authors:** 


### THE WESTERN JOURNAL.

—

THIRD SERIES.—VOL. I.—NO IV.

—

LOUISVILLE, APRIL 1, 1848.

---

### PROGRESS OF THE USE OF CHLOROFORM.

While the instances in which Chloroform has been used with safety and the most perfect success are counted now by thousands, we have to record at least one case in which there is good reason to believe that it caused the death of an individual. This case, reported by the editors of the Western Lancet, will be found in another department of this Number, and will not fail to command the attention of the reader. Our opinion is that this result ought not to have occurred from the use of the agent. It seems, in the first place, that the woman breathed the vapor from an instrument, which had the effect of excluding the atmosphere to a great extent. This was wrong, for it has been found that asphyxia is apt to take place when either chloroform or ether is thus administered. In the next place, the professional gentlemen do not appear to have watched the effects of the agent upon the pulse; and then, when asphyxia did occur, they left her sitting in the operating chair, instead of placing her at once in the recumbent posture. Of course, it is impossible to say, that, under these altered circumstances the catastrophe would not have happened, but it appears highly probable to us that it would not.

We find another fatal case from the use of chloroform reported in a newspaper, and although it requires confirmation, we deem it proper to quote it, that, as we have borne strong testimony to the virtues of the new anæsthetic, the reader may have likewise whatever testimony there may be against it.

"A young lady, daughter of Mr. MacDonald, a baker in Catharine street, New York, recently met her death in the most awful manner, from the use of this now fashionable but most dangerous preparation. About three weeks ago, the ether was employed to allay the tooth-ache; but subsequently the sufferer was supposed to die, from what cause does not appear. The apparent death, however, was only a trance, or protracted swoon; for, on opening the coffin a day or two ago, the unfortunate girl had turned round upon her face, and in her agony and desperation had actually destroyed two of her fingers, on recovering from her temporary death by ether. The Coroner's investigation should elicit the fact as to who prescribed a remedy which produced this most frightful result."

We will only add, at present, that, since our last report on this subject, chloroform has been administered by our professional friends, in this city, in a variety of cases, and, in all, with satisfactory results; and that our own experience with it has served to heighten our estimate of its value. Professor Miller has just told us that he gave it to a lady in labor a few nights ago with the most pleasing effects. The lady was of very full habit, and the Doctor hesitated about using an agent which was supposed to favor convulsions and apoplexy, but she was urgent, and he yielded to her importunities. She declared after the child was born, that although she was conscious of its birth, she felt no pain, and that chloroform was the greatest discovery in the world. Among other applications, we gave it to a little boy suffering with ear-ache, a few evenings since, and were gratified to see him, after a few inhalations, fall into a quiet, natural sleep. He was relieved of the pain, which was probably neuralgic.

Practitioners, however, cannot be too cautious in the use of an agent of so much power. The state of the pulse and of the respiratory function ought to be watched incessantly, and the first indications of an approach of asphyxia met by the appropriate remedies.

